# A qualitative study of cancer clinical trial network consumers’ acceptability of the modular approach to patient-reported outcome measurement: how much of it is “common sense”?

**DOI:** 10.1186/s41687-026-01045-w

**Published:** 2026-03-25

**Authors:** Carrie-Anne Ng, Mariana Sousa, Tiffany Patterson, Brendan Mulhern, Tim Luckett

**Affiliations:** 1 Centre for Health Economics Research and Evaluation, Faculty of Health, University of Technology Sydney, Sydney, NSW Australia; 2 Cancer Quality of Life Expert Service Team (CQUEST), University of Technology Sydney, Sydney, NSW Australia; 3 Improving Palliative, Aged and Chronic Care Through Clinical Research and Translation (IMPACCT), Faculty of Health, University of Technology Sydney, Sydney, NSW Australia; 4 School of Nursing and Midwifery, Western Sydney University, Sydney, NSW Australia; 5 Australian Centre for Integration of Oral Health, School of Nursing & Midwifery, Western Sydney University, Sydney, Australia; 6 Ingham Institute for Applied Medical Research, Sydney, Australia

**Keywords:** Modular approach, Patient-reported outcome, Response burden, Clinical trial

## Abstract

**Background:**

There is growing interest in customising patient-reported outcome measures (PROMs). This involves selecting specific domains (i.e., subscales) that focus on key health-related quality of life (HRQoL) issues most relevant to a particular study’s context, referred to as the *modular approach*. Despite available recommendations from international stakeholders and PROM developers, the modular approach has not been widely adopted in cancer clinical trials. This qualitative study explored the acceptability of the modular approach to administering PROMs from the perspectives of cancer consumers.

**Methods:**

Consumers (patients, family members and/or caregivers) who have experience with cancer were recruited through cancer clinical trial networks in Australian and New Zealand. Data were collected through online focus groups. Interview probes and analysis were guided by Sekhon et al.’s Theoretical Framework of Acceptability.

**Results:**

Ten consumers, all with prior experience in clinical trial design, participated across four focus groups. The acceptability of the modular approach was discussed in reference to four themes: (1) minimising respondent burden is not simply about shortening PROMs; (2) competing priorities of reducing burden, preserving PROM measurement properties and assessing breadth versus depth of issues; (3) new strategies are needed to improve PROM relevance; and (4) who should select the domains, and how? Acceptable features of the modular approach included its potential to increase the relevance of questions, minimise duplication, and allow for more in-depth assessment of priority HRQoL issues. Participants also believed that involving consumer representatives in domain selection could alleviate the burden on researchers during trial design and were supportive of giving individual clinical trial patients the option to self-select domains that are personally meaningful.

**Conclusions:**

Consumers were generally supportive of using the modular approach in administering PROMs in clinical trials, while also identifying ways to strengthen its acceptability. These included the need for clearer consensus and guidance on what constitutes a well-justified selection of domains and further personalisation of PROM domains (e.g., through branching questions). These insights can help inform regulatory agencies and other stakeholders about consumer needs and highlight the support needed if there is to be a paradigm shift in trial design towards tailored PROM administration.

**Supplementary Information:**

The online version contains supplementary material available at 10.1186/s41687-026-01045-w.

## Background

There is growing concern among international stakeholders that the cancer clinical trial community has defaulted to using specific full-length patient-reported outcome measures (PROMs) which may not adequately capture key domains of health-related quality of life (HRQoL) relevant to specific studies, contexts or patient populations. Additionally, they may include irrelevant domains, posing ethical concerns by unnecessarily burdening unwell patients [1–3].

In response, stakeholders have recently published position statements regarding a *modular approach*, where only a subset of domains (i.e. subscales) from a multi-domain PROM are administered [4, 5]. This approach has been proposed, through an international Delphi study, as a way to reduce respondent burden by assessing only the most relevant domains at key timepoints [6]. However, its effectiveness in reducing burden is unclear, and several key considerations remain [7]. While the modular approach offers greater flexibility in PROM assessment, it may miss unexpected HRQoL issues and hinder comparability with studies that have used the full instrument. Additionally, greater effort is required from researchers to clearly define what needs to be measured, ensuring that outcomes reflect the likely benefits and disadvantages of the treatment under investigation, and that the most important constructs are assessed in an even-handed way across comparators.

Current recommendations on the modular approach have largely adopted a top-down perspective, aimed at promoting strategic thinking for the use of PROMs across clinical trials. While position statements have included representatives from the patient advocacy organisations, much of the discourse has been driven by the views of PROM developers, health technology assessment bodies, regulatory agencies, and psychometric experts [4, 8]. However, the modular approach has yet to gain traction in cancer clinical trials, and its acceptability among certain stakeholder groups has not yet been explored.

Given that the most important rationale for the modular approach is to reduce burden on trial participants while maintaining measurement validity, it is incumbent upon the field to canvass the views of people living with cancer, their family and carers – collectively referred to as consumers (the preferred term by the Australian Commission on Safety and Quality in Health Care [9]). Indeed, there is growing recognition of the value of partnering with consumers in health research [10], with consumer involvement in PROM design now considered fundamental [11]. In Australia, cancer clinical trial networks bring together clinicians, health professionals, researchers, and consumers to collaboratively design and conduct cancer clinical trials, facilitating both peer- and consumer-review to support the development of high-quality clinical trials [12]. Gaining insight from consumers involved in such networks can help identify areas where further support or guidance is needed to facilitate the uptake of a tailored approach to PROMs. Therefore, the aim of this qualitative study was to explore the acceptability of the modular approach from the perspectives of consumers from cancer clinical trial networks.

## Methods

### Study design

A qualitative approach was used to gain an in-depth understanding of the acceptability of the modular approach from the perspectives of cancer consumers. The study was conducted between November 2024 and April 2025 and received ethical approval from the University of Technology Sydney Human Research Ethics Committee (ETH21-6090). All participants gave informed verbal consent. Reporting adheres to the COnsolidated criteria for REporting Qualitative research [13].

### Participants

Eligible participants were adults (aged ≥ 18 years) who were patients, family members and/or caregivers with experience with cancer. Participants were excluded if they were unable to provide informed consent or participate in the focus groups in English. Recruitment was conducted by inviting expressions of interest via a brief online form hosted on the Qualtrics survey platform, where individuals provided their contact details. The study was advertised in newsletters distributed by the Cancer Australia-funded Quality of life Technical Service, reaching subscribed members of the fourteen Australian and New Zealand multi-site collaborative cancer clinical trials groups, also funded by Cancer Australia. In addition, several of the trial groups disseminated invitations through their standard communication channels, including newsletters, member portals and social media (e.g., tweets), targeting either their broader membership or dedicated consumer advisory panel. Recruitment also occurred through announcements at relevant meetings of some clinical trials groups. Due to the recruitment methods used, the number of individuals invited but who did not express interest in participating could not be recorded to estimate a response rate. Consumers were offered an AUD50 voucher for their participation.

Convenience sampling was used and recruitment continued until code saturation was reached (i.e. no substantially new issues identified with additional focus groups) [14]. Given the narrow aim of the research and high likelihood of recruiting consumers who were experienced in clinical trial design, we anticipated conducting 3 to 6 focus groups [14, 15].

### Data collection

Focus groups were conducted via video conference (Zoom), audio-recorded and transcribed. Each focus group was facilitated by one (C.N.) or two (T.P. and C.N.) researchers. C.N. is a female health economist (PhD) with experience in qualitative research and in the use of HRQoL PROMs in cancer. T.P. is a female dietitian and lecturer (PhD) also with experience in qualitative research. The interviewers had no prior or ongoing relationships with any of the participants. Participants were informed that the researchers were not involved in their healthcare and were aware of the research’s purpose. To our knowledge, no individuals other than the participants and researcher(s) were present during the focus groups.

The topic guide is provided in Supplementary 1. Focus groups began with a brief introduction outlining the purpose of the study, establishing ground rules and obtaining verbal consent. Participants were then invited to introduce themselves to help establish rapport. The interviewer then delivered a brief presentation introducing the modular approach. Participants were introduced to the concept of HRQoL assessment using the European Organisation for Research and Treatment of Cancer (EORTC) QLQ-C30 questionnaire and its scoring as an example, selected because it is the most widely-used PROM in cancer clinical trials [16]. The primary rationale for the modular approach was explained to be reducing respondent burden through improving the relevance of questions by tailoring them to the specific clinical trial population and treatment. A scenario involving the EORTC QLQ-BN20 brain tumour module [17] was described, in which certain domains were removed based on the hypothesis that, given the tumour’s location in the brain in the target trial population, these domains were unlikely to be affected. The QLQ-BN20 was selected because primary brain tumours symptoms can be focal in nature, making the hypothesis relatively understandable.

A semi-structured approach was taken to questioning, beginning with an open question inviting participants’ views on the modular approach in general. Once unprompted issues were exhausted, the researcher(s) asked questions grouped according to the seven constructs of the theoretical framework of acceptability proposed by Sekhon et al. (2017) [18]. These constructs are affective attitude, burden, perceived effectiveness, intervention coherence, self-efficacy, opportunity cost and ethicality. As the framework was originally designed in the context of healthcare interventions, adaptations were made to explore participants’ perspectives either as consumer representatives contributing to the design of cancer clinical trials or clinical trial participants being asked to complete PROMs in which the modular approach was applied (Table 1).

**Table 1:** Adapted Sekhon’s theoretical framework of acceptability

Component constructs	Original definition	Adapted definition
Affective attitude	How an individual feels about the intervention, prior to taking part	How an individual feels about the use of the modular approach in PROMs
Burden	The perceived amount of effort that is required to participate in the intervention	The perceived amount of effort that is required to (A) apply the modular approach during trial design and (B) complete PROMs in which the modular approach was applied
Perceived effectiveness	The extent to which the intervention is perceived to be likely to achieve its purpose	The extent to which the modular approach is perceived to be likely to achieve its purpose
(Intervention) Coherence	The extent to which the participant understands the intervention and how it works	The extent to which individuals understands the modular approach and how it works
Self-efficacy	The participant’s confidence that they can perform the behaviour(s) required to participate in the intervention	The individual’s confidence that they can (A) apply the modular approach to PROM(s) during trial design and (B) complete a PROM in which the modular approach was applied.
Opportunity cost	The extent to which benefits, profits, or values must be given up to engage in the intervention	The extent to which benefits, profits, or values must be given up when a modular approach is used.
Ethicality	The extent to which the intervention has good fit with an individual’s value system	The extent to which the modular approach has good fit with an individual’s value system.

Focus groups concluded with an opportunity for participants to raise any additional issues not previously discussed. In addition, participants were asked about their involvement in clinical trials or clinical trial design, diagnosed cancer type, and demographic information. The researchers made field notes on observations during and after the interviews. No repeat interviews were carried out. Transcripts were imported to NVivo version 12 software for management and analysis.

### Analysis

Data were analysed using an integrated approach that involved both inductive and deductive coding [19]. Initially, transcripts were coded inductively by C.N. and T.P. to ensure that insights shared by participants were captured regardless of their fit to the acceptability framework. These data were charted to identify patterns and form descriptive categories. Coding was initially conducted after the first two focus groups and subsequently after each additional group, allowing emerging issues to be revisited in the next round of data collection for verification and further refinement. Coding saturation was monitored during analysis through ongoing review of the code set and team-based discussions to ensure that any additional data confirmed rather than extended the code set. These codes were then deductively mapped onto the adapted acceptability framework, and any codes that did not easily fit were identified. Broad, inductively derived themes were also identified and subsequently refined through discussion among the authors (C.N., T.L., B.M.). Participants were invited to review a one-page summary of the overall study’s findings and disagree or suggest refinements if necessary.

## Results

Fifteen consumers expressed interest in the study, of whom 10 participated in 4 focus groups (*n* = 2–3 per group). All participants were members of consumer advisory panels affiliated with Australian/New Zealand clinical trial networks at the time of being interviewed. Table 2 presents the characteristics of the participants. Focus groups lasted between 40 and 57 min (mean = 50 min). No new codes emerged in the final focus group.

**Table 2:** Characteristics of consumer participants (*n* = 10)

	Participants
Gender	
Male	4
Female	6
Age	
45 to 54	2
55 to 64	2
65 to 74	6
Place of residence	
Major City	7
Inner/Outer Regional	2
Rural	1
Country of birth	
Australia	5
New Zealand	1
England	3
USA	1
Highest level of education	
Year 11 and below	1
Diploma/advanced diploma	1
Bachelors or honours degree	5
Postgraduate degree (Masters/doctorate)	3
Consumer group	
Patient with experience with cancer	7
Family member and/or caregiver of a person with experience with cancer	3
Diagnosed cancer type^1^	
Breast	2
Bowel	1
Gastric	1
Leukaemia	1
Lung	1
Pancreas	1
Prostate/testicular	1
Thyroid	1
Ovarian	1
Previous experience of being a clinical trial participant	
Yes	4
No	6

^1^ Participants were asked to self-report what cancer they or their family member/care recipient were/was diagnosed with. If there was a diagnosis of more than one cancer, the cancer that was diagnosed most recently was reported

Overall, participants gave the impression they understood the primary purpose and basic principles of applying the modular approach to PROMs, and there were very few instances where they felt unable to offer an opinion. There was broad acceptance that the modular approach would be advantageous when administering PROMs in clinical trials, with some describing it as “common sense” (C5) or “making sense” (C6). When unprompted, participants adopted either the perspective of consumer representatives involved in clinical trial design, or that of trial participants completing PROMs.

Participants’ reflections on the acceptability of using the modular approach were summarised according to four main themes:

1. Minimising respondent burden is not simply about shortening PROMs.
2. Competing priorities of reducing burden, preserving PROM measurement properties and assessing breadth versus depth of issues.
3. New strategies are needed to improve PROM relevance.
4. Who should select the domains, and how?

These themes were mapped to all seven component constructs of the theoretical framework of acceptability (Table 3).

**Table 3:** Findings related to the modular approach mapped to the adapted theoretical framework of acceptability by Sekhon et al. (2017)

Component constructs	Mapped theme	Description
Affective attitude	1, 3	Positive attitudes toward the use of the modular approach
	2	Concern that important domains may be excluded
	4	Some expressed caution, emphasising the importance of consulting with consumers to apply the modular approach appropriately
Burden	1, 2, 4	A worthwhile endeavour despite greater effort needed to choose appropriate domains
	1	Tailored questionnaire has greater relevance and less duplicative questions.
Perceived effectiveness	1	Potential to improve data quality through reduced burden
	2	Questions focused on relevant domains can generate more meaningful data in a shorter time. However, the comparability of PROMs may be limited.
	3	Flexibility enables assessment of both breadth and depth of issues at appropriate time points
	4	Choices of domains based on research hypothese or common issues seen as sensible
Coherence	2	PROM measurement properties could be affected by modifications
	4	Some uncertainty about what constitutes a relevant and/or important domain
Self-efficacy	4	Clearer guidance about the appropriate use and implications of the modular approach when developing a PROM strategy was sought
	3, 4	Clinical trial patients should be provided a choice to self-select relevant and/or important domains
	3, 4	Providing explanations for why each domain is assessed can facilitate PROM engagement
Opportunity cost	2	Important domains may be excluded
	2	Removal of questions from a PROM may result in the loss of contextual information that assist with PROM completion
	4	Current novelty of the modular approach may limit its acceptability among stakeholders, and there was a need to build its evidence base or establish consensus and detailed guidance to support its adoption
Ethicality	1	Acknowledges and respects clinical trial patients’ time, especially when they are unwell
	3	Potential to improve clinical trial access to cultural and linguistically diverse groups
	4	Concern about potential bias when investigator select the domains themselves

Note: Mapped themes were: (1) Minimising respondent burden is not simply about shortening PROMs; (2) Competing priorities of reducing burden, preserving PROM measurement properties and assessing breadth versus depth of issues; (3) New strategies are needed to improve PROM relevance; and (4) Who should select the domains, and how?

### Theme 1: Minimising respondent burden goes beyond simply shortening PROMs

Participants generally viewed the modular approach’s potential to reduce respondent burden positively, seeing it as a way to support clinical trial participation and retention, and improve data quality, in accordance with the ethical principles of patient-centred research.

It’s about time saving. Because – for some people – that’s why they either don’t participate in clinical trials, or they might drop out because they feel that it’s too much of a burden on their time. (C2).

While the idea of administering fewer questions was welcomed, some questioned whether “people actually find long questionnaires problematic” (C6). Individuals’ perceived tolerance for PROM burden varied, with a few participants seeing it as necessary or to be expected as part of being on a clinical trial:

You’re putting your hand up… you know that there may be questions you don’t like. (C7).

Nonetheless, there was strong indication throughout all discussions that the real value of the modular approach lies in asking more relevant and non-repetitive questions.

I’m trained in science, so I wouldn’t mind – if I’m going to join a clinical trial – a long questionnaire… I would not be troubled by it, but I might be irritated if there were irrelevant questions. (C6).

Most participants also agreed that clinical trial research will benefit from improved data quality, driven by “more sophisticated and attentive engagement” (C3) with PROMs and reduced frustration on the part of respondents. In one discussion where the interviewer introduced the idea of using the modular approach to tailor questions at specific PROM assessment time points, participants responded favourably, with one reflecting on this through an analogy:

It’d be like the nurse coming in and taking your vitals every half hour. You don’t get a full body scan every half hour - it’s what’s appropriate to do on a more regular basis. (C5).

Importantly, several participants described the modular approach as “ethical because it’s relieving the patient of having to go through unnecessary paperwork” (C5) or as a way to help ensure that “their time is not being effectively wasted” (C3), especially when they are undergoing intensive treatment such as chemotherapy.

“Chemo affects people’s brain function more than others. We would hope that a lot of research that is coming out would hopefully be in consideration of the treatment that they’re giving.” (C4).

### Theme 2: Competing priorities of reducing burden, preserving PROM measurement properties, and assessing a breadth versus depth of issues

Discussions about the benefits of reducing respondent burden were frequently accompanied by concerns about modifying PROMs. Several participants questioned whether such modifications might affect the measurement properties or “integrity” (C4) of the questionnaire compared to the standard form, even though this was not brought up in the introductory presentation. Some also raised the possibility that altering PROMs could “challenge the outcomes of studies in terms of validity and reliability” (C1) or “skew your results” (C9).

A few participants also expressed concern that respondents could lose contextual information or “framing” (C10) of questions, if related questions were removed from a PROM, or if questions from multiple PROMs were combined.

It’s like scrambled eggs – you’ve got all these different questions under one banner, and they’re not defined… You’ve got to ‘modularise’ them out into different elements… Kind of like, ‘So this is going to be my answer, based on my questions on pain, or based on my questions on social interaction.’ (C1).

There were mixed views on whether reducing burden should be prioritised as a “default” (C5) across all clinical trials, or on a case-by-case basis, such as in trials with intensive treatment like surgery or chemotherapy.

It’s a tough one, because all treatments are different for different people. I think the main thing is that researchers themselves should think about when they’re administering these questionnaires when appropriate. (C4).

Participants had an impression that PROMs used in clinical trials tended to be too broad, describing them as “level-one questions” (C8) to acquire “only a primary level of understanding” (C3). Nonetheless, some participants acknowledged the value of a “shotgun approach” (C7), as it could help avoid “missing out on certain information” (C2). There was agreement that these questions should be presented appropriately to respondents.

If it was too trimmed down, I would think it’s not gathering all the information I would like to provide… I think that the more questions there are, when they are appropriately categorised and explained, provide better information for researchers to work with. (C1).

Some participants understood that regardless of how thoughtfully domains were selected, some information will inevitably be missed, which could be an inherent limitation of PROMs.

You will never cover them all. There’ll be something that pops up somewhere that you didn’t think of. (C8).

Back at the very start (of PROM development), somebody had to decide. They already made that presumption about the questions you’re not going to ask. (C7).

Other participants felt that using the modular approach to narrow the focus to specific domains could yield richer, more meaningful data, comparable to insights from qualitative research, without requiring the same level of resources.

These surveys that are very clunky and paper-based don’t lift from the page in the form that, for example, a medical consultation would. Less is more, if targeted questions are asked… That would enable the objectivity of the data to be preserved, and I think that is obviously valuable. (C3).

Across all focus groups, participants spontaneously emphasised the importance of including open-ended questions as a “catch-all if the study participant thinks there’s something you need to know” (C9), or to provide more contextual information about previous responses.

### Theme 3: Encouraging the development of strategies to improve PROM relevance

Almost all participants spontaneously suggested strategies for applying the modular approach in PROM administration. In some discussions, the use of branching questions was suggested to first determine whether a patient had an issue, and, if so, to ask for more details about their experience. Participants agreed on the importance of allowing patients to choose the domains they find are relevant, noting that “every person is different” (C10).

Ask participants if they think the included modules fit their responses, and if they would like to include other information in the form of other modules. (C1).

You give the person a form, and you say, ‘could you have a look at this and answer the questions that you feel are relevant?’ (C8).

A few participants believed that self-selected modules would help facilitate engagement, especially if they were followed-up on.

People’s overall enthusiasm for participation is increased because people would feel that they were being listened to… if follow-up questions are more targeted (C3).

There was strong agreement that explaining to trial patients why certain questions are being asked is essential for promoting engagement with PROMs. Some participants further suggested that this information should be provided for each HRQoL construct being assessed and contextualised within the specific clinical trial.

As long as you’re upfront and explain to the patient, say, ‘We know there’ll be times you’ll be experiencing a lot more pain than others, and so, we would like to question you about your levels of pain more frequently over the course of this trial.’ (C5).

In the context of the modular approach, this accompanying explanation could be reduced if the chosen domains were tailored.

You’re still going to need to explain it to them. I’d rather explain (why there are questions for) 7 criteria, than 11 criteria. (C9).

Improving PROM relevance to patients from cultural and linguistically diverse (CALD) backgrounds, or those with cognitive impairment was mentioned as important in two focus groups, although it was unclear how the modular approach could be used in these contexts.

For a lot of trials involving people who English is not a first language, I don’t see how things like that fit into the modular approach, but maybe you need to bring something like that in, because there’s a vast number in our multicultural society that may miss out on getting access. (C10).

### Theme 4: Who should select the domains, and how?

Participants were generally supportive of researchers applying the modular approach to enable more thoughtful inclusion of PROMs in trial design.

The modular approach will force the researcher to turn their minds to the questionnaire and its use and relevance rather than tacking on the standard form (C6).

However, caution was expressed about researchers being the sole decision-makers, with participants noting the consumers could help share the additional burden involved in making appropriate selection – especially if the panel was “truly representative” (C9) of the clinical trial context.

It’s really good to get (consumers) involved right from the design stage. Otherwise, it becomes a little biased and could end up being a burden on the researcher trying to figure out what modules to use. They might just end up lumping a whole lot of questions for the sake of ticking a box and saying, ‘Yes, we have a quality-of-life survey in there.’ … The patient’s voice would definitely help any researcher. (C4).

Participants agreed that clear communication between researcher and consumers in a “two-way partnership” (C8) will help facilitate these discussions.

…explain to the consumers why these specific questions are in there. It’s really an exercise for the researcher to know their specific topic and tailor their questions accordingly. (C9).

Clear research hypotheses, or “looking at what we need to get out of this” (C10), were described as an important starting point in selecting domains. Practical suggestions included presenting sample questionnaires to a study’s consumer advisory panel to clarify “what exactly they’re asking” (C4) and educating consumers on which types of questionnaires are considered best practice in specific trial settings. Participants also believed that choosing modules based on issues that “come up more regularly” (C7) was a sensible strategy. However, some participants sought clarification over what constitutes a relevant domain, or the reasoning behind selecting one questionnaire over another.

I have difficulty understanding the idea of making assumptions when you’re talking about questions… The problem I have with all of this is the issue of relevance – it’s for the person who’s answering the question to decide, not the person who’s asking. (C8).

Indeed, most participants were confident that clinical trial patients will be able to choose self-select modules that they felt were relevant to them on an ad-hoc basis. In one focus group, however, participants expressed caution because “sometimes patients are very reluctant to be honest about how bad they are because they don’t want to cause concern” (C9).

## Discussion

Mapping our findings to the acceptability framework highlighted key facilitators and barriers of the modular approach, along with potential opportunities for implementation. Our findings indicate that features of the modular approach considered acceptable to consumers included its potential to reduce respondent burden by increasing the relevance of questions, minimising duplication, and enabling more in-depth assessment of important HRQoL issues. Indeed, it is increasingly recognised that questionnaire length is not the primary contributor to burden [6, 20]. In our study, participants also strongly supported the idea that domain selection should be a collaborative process between clinical trial researchers and consumers, while still offering individual trial patients the opportunity to self-select domains that are personally meaningful.

Similar findings regarding patients’ preferences for PROMs were reported in a meta-synthesis of 14 studies [21], which found that patients preferred questions relevant to their health problems, the inclusion of open-ended questions, and in-depth items to capture fluctuating symptoms and nuances in HRQoL issues. Patients have also likewise expressed a desire to understand the purpose of PROMs [22]. In our study, consumers suggested that patient engagement could be improved by tailoring explanations to the specific trial context or by communicating hypothesised effects. For example, a previous study found that informing patients about the reasons for follow up when they did not complete PROMs within seven days, including the need to assess potential declines in HRQoL, improved PROM completion [23]. As minimal guidance or interpretation of items is typically recommended to maintain standardisation in clinical trials [24], any accompanying rationales should remain general. For example, patients could be informed that the included PROMs assess HRQoL issues identified by clinicians and consumers as potentially impacted by the disease and treatment within the clinical trial.

Our study also suggests that providing accompanying information to define specific HRQoL constructs may facilitate PROM completion. This additional context could help mitigate concerns about item-order effects associated with the modular approach, in which the validity of domains could be compromised if responses are influenced by preceding questions [4, 8]. Some PROMs may be more susceptible to this effect than others. In the fatigue-specific EORTC QLQ-FA12, questions of the physical fatigue and cognitive fatigue domains provide descriptions about fatigue (e.g., “lacked energy”) or contextual information (e.g., “Did tiredness interfere…”), while questions from the emotional fatigue domain are less explicitly linked to fatigue (e.g., “Did you feel discouraged?”) [25]. In isolation, these emotional fatigue questions could be interpreted more broadly as assessments of emotional functioning and could benefit from contextual information. However, PROM instructions are typically created alongside questions during development, and are collectively assessed for face validity and other psychometric properties [26]. Nonetheless, the impact of defining key constructs through more standardised means to support PROM completion and the use of the modular approach may warrant further investigation.

The self-selection of domains by individual clinical trial patients was identified as a promising application of the modular approach. This concept has been explored previously through methods like goal attainment scaling [27] and the Schedule for the Evaluation of Individual Quality of Life (SEIQoL) [28], which use interviews to allow patients to freely nominate important aspects of HRQoL. Similarly, self-administered PROMs like the Measure Yourself Medical Outcomes Profile [29] or Write In three Symptoms-Problems [30] ask patients to define and rate symptoms that bother them the most. However, these tools are often criticised for their lack of score comparability and difficulties in evaluating psychometric properties. The modular approach could address these limitations by presenting domains that have already been validated and used in past research. It could also complement open-ended questions recommended in early-phase research to capture unexpected or previously unidentified adverse events [31]. For example, a system was developed where patients’ open-ended responses triggered relevant options to be appear in a dropdown menu, allowing them to select symptoms from the Patient-Reported Outcomes version of the Common Terminology Criteria for Adverse Events (PRO-CTCAE) item library for rating [32]. Our focus groups also suggested that following up on patients’ prior choices may also improve PROM engagement and provide the advantage of longitudinal data. However, as indicated in our discussions, relying heavily on this self-selection strategy could introduce bias, especially if patients are not able to sufficiently reflect or self-identify the important or relevant domains at the time of PROM administration [21], or withhold concerns for fear that doing so may lead to treatment modifications [33]. Nonetheless, implementation of these strategies in early-phase trials as a complement to pre-selected PROMs and/or domains, could help estimate the nature or magnitude of such effects to inform PROMs and sample size for future research.

Another potential area of application of the modular approach raised during the focus groups is improving clinical trial access for CALD populations, although how this could be achieved was unclear to participants. If the selection of PROM domains is guided using conceptual frameworks and core PRO sets [7], researchers can prioritise PROMs with available translations and, where possible, adequate cross-cultural validation. This approach can help to preserve certain psychometric properties, including content validity, and be beneficial in situations where developing or validating bespoke PROMs may be challenging [34]. Future studies may develop and validate PROM strategies using the modular approach for CALD groups where no existing PROM adequately captures a full range of relevant issues, ensuring that concepts of interest are appropriately represented with input from consumers.

All participants were members of consumer advisory panels, which are typically established in clinical trial networks to ensure the consumer perspective is incorporated into the planning and conduct of trials [10, 35]. Drawing on their involvement, they identified areas where additional support is needed to assist consumers contributing to clinical trial protocols that include PROMs. A knowledge transfer tool has previously been co-developed following the Standard Protocol Items: Recommendations for Interventional Trials (SPIRIT-PRO) extension guidance, which includes considerations of whether a PROM is “relevant for the patient group”, “appropriate” and “acceptable” [36]. Our findings suggest that more support is needed regarding these criteria, which would also benefit investigators, especially given the wide availability of PROM domains for the measuring same HRQOL concept.

Participants in our study found the introductory presentation in this study about the modular approach to be helpful in helping in building their understanding. We used the example of a brain tumour trial population with focal symptoms to explain why certain domains might be excluded as this appeared straightforward. However, we acknowledge that this example may have been too unfamiliar to effectively elicit opinions on domain selection [37]. In future exercises, presenting a variety of rationales may better capture nuances in perspectives. Evidence demonstrating the successful use of the modular approach in clinical trials would also likely support this process.

Although recruitment was not specifically limited to consumers from consumer advisory panels, the final sample consisted of such individual with considerable experience in clinical trial design. This was a strength, as participants were familiar with the use and integration of PROMs in trial design and readily grasped the concept of the modular approach. However, their perspectives may not represent the broader patient population, and we also did not have representatives of CALD populations, or of specific conditions (e.g. rare diseases). Including individuals with less experience or from diverse populations could have highlighted additional identify areas of support for uptake of the modular approach [38].

## Conclusion

Consumers agreed that the modular approach offers advantages when administering PROMs in clinical trials, particularly for intensive treatments associated with high patient burden, and focusing assessment to relevant domains at appropriate time points. They emphasised the importance of involving consumers in decisions about which PROM domains to include or exclude. The less acceptable aspects of the modular approach revealed several areas for future research if there is to be a paradigm shift toward tailored PROM administration. These include offering patients the opportunity to self-select important domains. Additionally, there is a need to provide clear guidance to both clinical trialists and consumers on what constitutes a well-justified selection of domains, while balancing competing priorities. These insights can inform regulatory agencies and key stakeholders to understand consumer needs and demands.

## Supplementary Information

Below is the link to the electronic supplementary material.

Supplementary Material 1

## Data Availability

The datasets generated and/or analysed during the current study are not publicly available due to the risk of re-identifying participants from whole transcripts.

## Abbreviations

PROMs: Patient-reported outcome measures
HRQoL: Health-related quality of life
EORTC: European Organisation for Research and Treatment of Cancer
CALD: Cultural and linguistically diverse
