# Supplementary material for: A qualitative study of cancer clinical trial network consumers’ acceptability of the modular approach to patient-reported outcome measurement: how much of it is “common sense”?
Source: J Patient Rep Outcomes. 2026 Mar 25;10:72. doi: 10.1186/s41687-026-01045-w (PMC13136423; doi:10.1186/s41687-026-01045-w)
Supplement: Supplementary file 1 — Supplementary Material 1 [file 41687_2026_1045_MOESM1_ESM.docx]

**Supplementary 1**

Topic guide for focus groups

| **Background / warm-up questions** | - Briefly describe your involvement with clinical trials / consumer advisory panel (if applicable). - Have you ever participated in a clinical trial?   - (If yes) Did you receive a questionnaire in a clinical trial asking you about your health?   - (If no) Have you ever received a questionnaire asking you about your health? - What kinds of questions were asked? - What did you think about the questionnaire? Do you think it could be improved? How? |
| --- | --- |
| **Introduce modular approach** | Introductory presentation:   - Introduce key terminologies (health-related quality of life (HRQoL), patient-reported outcome measures (PROMs)) - Introduce EORTC QLQ-C30 questionnaire and its scoring - Describe how the modular approach is applied, and its rationale – primarily aiming to reduce respondent burden and improve the relevance of questions by tailoring them to the specific clinical trial population and treatment. - Describe scenario using the EORTC QLQ-BN20 brain tumour module   - Introduce QLQ-BN20 questionnaire and its scoring   - Scenario: An investigator selects relevant domains from the QLQ-BN20 for his clinical trial based on prior research, consumer input and clinical experience. It was hypothesised that, due to the location of brain tumour in the specific clinical trial population, visual disorders, communication deficits, and problems with bladder control were unlikely to be affected. The treatment or disease were also not expected to result in hair loss. Therefore, these four domains were removed for purposes of the hypothetical trial.   Follow-up questions:   - Do you have any questions about this? - What are your thoughts about using the modular approach in cancer clinical trials? |
| ***1. Affective Attitude:*** | - What do you like or dislike about it? |
| ***2. Burden*** | One of the reasons of using the modular approach is to reduce participant burden by shortening questionnaires and removing less relevant questions.   - How much priority do you give to reduce burden? - In what situations do you think it won’t reduce participant burden? - Can you think of other ways we can reduce the burden of questionnaires? Do you think these ways are a better alternative to the modular approach? |
| ***3. Perceived Effectiveness*** | - How effective do you think the modular approach will be in improving the relevance of questions? - Are there certain areas or groups of patients do you think the modular approach will be more effective for? - In what situations will it not work? |
| ***4. (Intervention) Coherence*** | - What things do you think need to done to use the modular approach appropriately? - Oftentimes, the clinical trial investigators are the one who decide what questionnaires to include in a trial. What do you think about that? - Data collected from questionnaires using a modular approach will be different than data collected using the whole questionnaire. Do you think this will be a big difference? |
| ***5. Self-efficacy*** | - If an investigator wants to use the modular approach in their trial and asks for your advice as a consumer representative, how confident are you in making sure it is done appropriately? What kind of things will help you in this process? - How confident are you in answering a questionnaire in which the modular approach was applied? |
| ***6. Opportunity Cost*** | - What are the barriers to using the modular approach? - Is there anything we are giving up so that we can use the modular approach? Are these things important? How? |
| ***7. Ethicality*** | - Do you think there are any ethical issues with the modular approach? - *If further prompting is required, ask:* When removing certain irrelevant questions from a questionnaire, we need to make assumptions that these questions are indeed irrelevant to patients. How much of an ethical problem will this be? |
| **Conclusion** | - Would you like to discuss anything important that we have not covered today? - Obtain additional demographic information - Thank participant for their time. |
